# Assessing small-mammal trapping design using spatially explicit capture recapture (SECR) modeling on long-term monitoring data

**DOI:** 10.1371/journal.pone.0270082

**Published:** 2022-07-05

**Authors:** Chase M. Freeman, Laureen Barthman-Thompson, Robert Klinger, Isa Woo, Karen M. Thorne

**Affiliations:** 1 U.S. Geological Survey, Western Ecological Research Center, Davis, CA, United States of America; 2 California Department of Fish and Wildlife, Stockton, CA, United States of America; 3 U.S. Geological Survey, Western Ecological Research Center, Sacramento, CA, United States of America; 4 U.S. Geological Survey, Western Ecological Research Center, Moffett Field, CA, United States of America; Museu de Ciències Naturals de Granollers, SPAIN

## Abstract

Few studies have evaluated the optimal sampling design for tracking small mammal population trends, especially for rare or difficult to detect species. Spatially explicit capture-recapture (SECR) models present an advancement over non-spatial models by accounting for individual movement when estimating density. The salt marsh harvest mouse (SMHM; *Reithrodontomys raviventris*) is a federal and California state listed endangered species endemic to the San Francisco Bay-Delta estuary, California, USA; where a population in a subembayment has been continually monitored over an 18-year period using mark-recapture methods. We analyzed capture data within a SECR modeling framework that allowed us to account for differences in detection and movement between sexes. We compared the full dataset to subsampling scenarios to evaluate how the grid size (area) of the trap design, trap density (spacing), and number of consecutive trapping occasions (duration) influenced density estimates. To validate the subsampling methods, we ran Monte Carlo simulations based on the true parameter estimates for each specific year. We found that reducing the area of the trapping design by more than 36% resulted in the inability of the SECR model to replicate density estimates within the SE of the original density estimates. However, when trapping occasions were reduced from 4 to 3-nights the density estimates were indistinguishable from the full dataset. Furthermore, reducing trap density by 50% also resulted in density estimates comparable to the full dataset and was a substantially better model than reducing the trap area by 50%. Overall, our results indicated that moderate reductions in the number of trapping occasions or trap density could yield similar density estimates when using a SECR approach. This approach allows the optimization of field trapping efforts and designs by reducing field efforts while maintaining the same population estimate compared to the full dataset. Using a SECR approach may help other wildlife programs identify sampling efficiencies without sacrificing data integrity for long term monitoring of population densities.

## Introduction

Obtaining accurate estimates of density and abundance is fundamental for quantifying and tracking wildlife populations for species conservation and management [1]. Managers are often faced with balancing robust field monitoring designs with the level of effort, especially for long-term monitoring programs that require a sustained level of staff and funding over time. Assessing long-term datasets can provide important insights on the efficacy of these field sampling methods and reveal potential improvements or time savings for wildlife monitoring. The use of small mammal traps is a key method for determining the presence and abundance of small mammals in habitats where animal detection may be difficult [2–4]. Small mammal trapping has been successfully used for decades to monitor populations [e.g., 5, 6] and inform conservation questions [e.g., 7–9]. For example, in south Africa trapping across functional groups provided information on impacts of agriculture practices adjacent to conservation lands [10], and Sherman and pitfall traps were effective in capturing small mammals in Madagascar to determine impacts from forest isolation and presence of wild boar and cattle [11]. Small mammal trapping has also provided data for deriving species richness, density, and population abundance estimates, which can then be used to evaluate climate-richness relationships [12] or related to habitat complexity, land use, and communities [e.g., 13–17]. The data can also be used to re-evaluate field efforts [18] and inform management and conservation goals [19]. Long-term population data can be especially valuable for assesing changes in populations, composition, community demographics, and local colonization/extinction rates [20, 21]. Thus, the effectiveness of conservation practices, management actions [22], and effects of landscape factors such as habitat disturbance can be determined through time [23].

A variety of live trapping methods are available to determine the presence or absence of small mammal species, including pitfall traps, cameras, and small enclosures (e.g., Sherman and Tomahawk live traps). But while many basic aspects of sampling have remained unchanged for decades, increased costs and logistical constraints have resulted in more scrutiny of trapping designs. A principal goal of most trapping studies is to ensure data are comparable across space and time while also taking into account level of effort, so trap number, arrangement (i.e., grid, transect, random, array), density (i.e., number of traps or spacing between traps), and duration (i.e., number of consecutive trapping nights) become very important considerations [24–27].

In addition to trapping design, a number of analytical approaches based on capture-recapture (CR) models have been developed over the last 50 years to estimate population size from live-trapping data [28–30]. The statistical foundations for estimating population size from CR histories were detailed by Otis et al. (1978), but estimating the effective sampling/trapping area, and thus population density, remained a significant challenge for many decades. Recently, spatially explicit capture-recapture (SECR) models have addressed this issue by incorporating movement and spatial distribution of captured individuals relative to trap locations to derive a detection function for making estimates of population density [31–33]. SECR models have many advantages compared to conventional CR models, with perhaps the most important being an objective determination of the area for estimating density [34–36].

Our objective was to use SECR modeling to evaluate different sampling designs with the goal of optimizing sampling effort while preserving data integrity and maintaining robust density estimates. We used a continuous 18-year salt marsh harvest mouse (SMHM; *Reithrodontomys raviventris*) CR dataset and modeled changes in grid size, trap density, and the number of consecutive trapping occasions to determine an optimal sampling design while maintaining acceptable precision of density estimates. We addressed two main questions: 1) How did grid size (trap number), trap density, and the number of trapping occasions affect density estimates? and 2) Could we decrease trapping efforts and still achieve the same population estimates?

## Materials and methods

### Study species

The salt marsh harvest mouse is endemic to the tidal marshes of the San Francisco Bay- Delta estuary in northern California, USA. The species’ historical tidal marsh habitat has been reduced by approximately 80% due to diking, draining, and filling for urban, industrial, and agricultural development [37], leading to its listing as endangered by both the federal and state governments [38]. The SMHM occupies tidal marsh, managed marsh, and adjacent habitat types that consist of wetland plant species that vary with salinities that range from freshwater to sea water. In tidal marsh, areas with mixed wetland plant species, such as fat hen (*Atriplex triangularis*), saltgrass (*Distichlis spicata*), Baltic rush (*Juncus balticus*), and Olney’s three-square bulrush (*Schoenoplectus americanus*), had greater SMHM densities compared to areas dominated by common pickleweed (*Salicornia* spp.) [39]. In tidal and managed marshes, both mixed-vegetation and pickleweed-dominated areas supported greater SMHM densities than upland habitats dominated by grasses [39]. Mixed vegetation including rabbitsfoot grass (*Polypogon monspeliensis*), fat hen, pickleweed, watergrass *(Echinochloa crus-galli*), and alkali bulrush (*Bolboschoenus maritimus*) may provide additional seed resources for foraging SMHM [40]. Within pickleweed-dominated tidal marshes SMHM appeared to be a habitat generalist and associated positively with larger continuous marsh patch sizes that were not bisected by levees or roads [41]. Habitat loss, degradation, and fragmentation as well as species interactions, competition, contaminants, disease, and climate related impacts such as sea-level rise, severe drought, and changes in heavy storms, are all challenges to SMHM recovery; however, no single driver of SMHM population densities has been identified and many data gaps remain [42]. A key component of addressing data gaps includes long-term monitoring, which needs to be optimized for staff effort in order to be sustainable. Long term density estimates of SMHM provide insight into environmental, landscape, and management variables that influence populations; thus monitoring their populations is a key priority in the conservation and recovery of the species [43–45]. Therefore, developing a standard trapping design protocol for SMHM is part of a species-wide effort to improve data consistency among management entities [42].

### Study site

**Fig 1:**
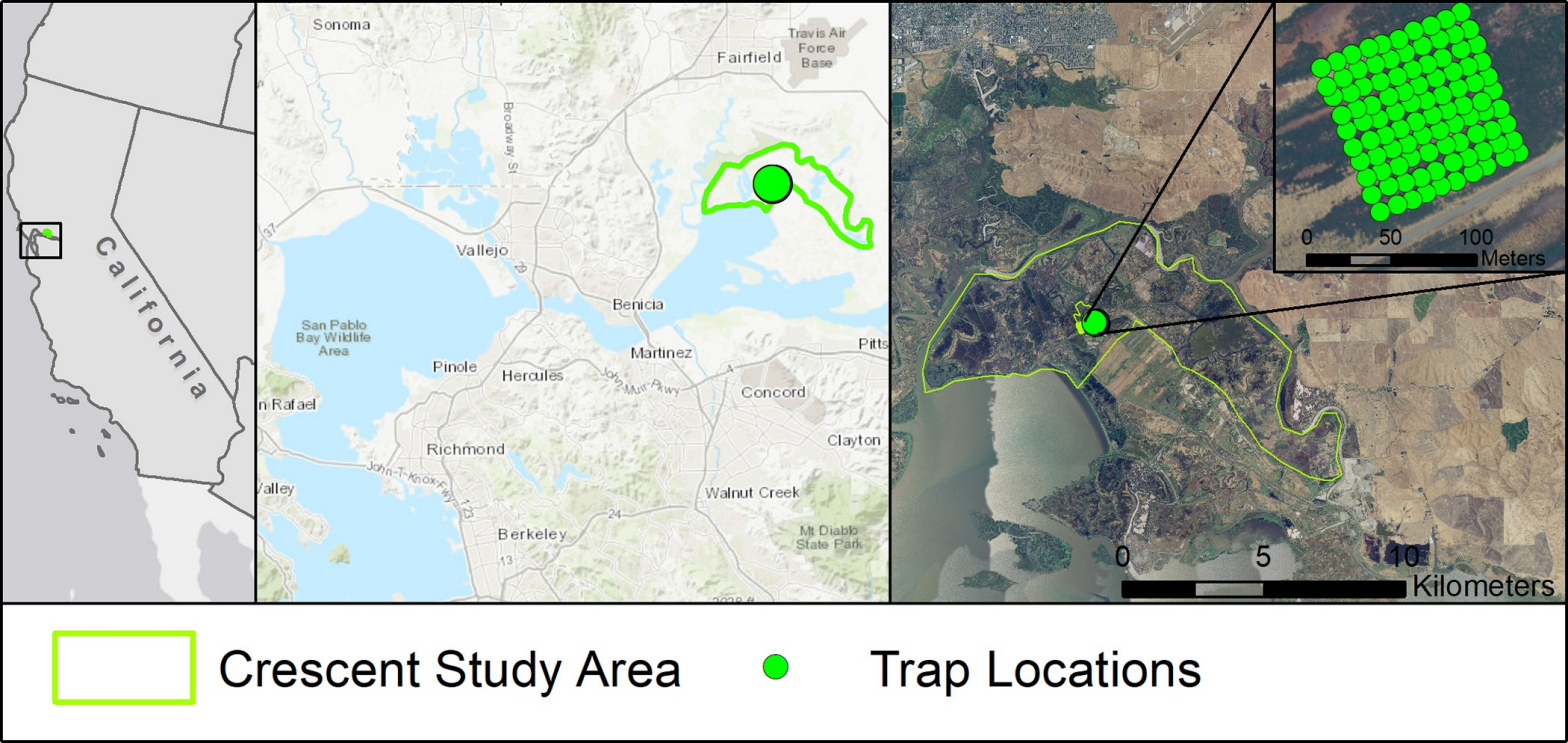
The study site is within a Suisun Bay marsh located in the eastern portion of the San Francisco Bay-Delta estuary, CA.

The study was conducted in the Suisun Marsh, Solano County, within the San Francisco Bay-Delta estuary located approximately 30 miles northeast of San Francisco, California (Fig 1). Historically, the area was a complex of sloughs, ponds, and tidal marshes. Presently the Suisun Marsh is a mosaic of diked marshes managed seasonally for migratory waterfowl and other wildlife with 6,300 acres of relatively unaltered tidal marshes, uplands, bays, and sloughs [37]. Annual SMHM monitoring has been conducted from 1998 to the present using live trapping efforts [42]. For this analysis we used survey data from one location within Suisun Marsh known as the California Department of Fish & Wildlife’s Grizzly Island Wildlife Area, Crescent Unit, a diked managed wetland area within Suisun Marsh (Fig 1). Vegetation within Crescent Unit, and specifically within the trapping grid, was dominated by pickleweed (*Salicornia pacifica*), but also included rabbitsfoot grass, brassbuttons (*Cotula coronopifolia*), and bare ground.

### Trapping

The trapping arrangement consisted of a square grid of 10 x 10 traps (total 100 traps) at approximately 10 m spacing intervals (9.43 meters exact spacing calculated by SECR package; Fig 1). Within an hour of sunset, Sherman live traps (7.62x7.62x25.4 cm; H.B. Sherman Traps, Tallahassee, FL) were baited and left closed on site for two nights to allow animals to become adjusted to the trap’s presence. On the third night the traps were opened at dusk and checked at dawn; all animals were processed (below), and traps were closed during the day. This procedure was repeated for three consecutive nights. Therefore, a single trapping event included repeated trapping over 4 consecutive occasions (nights), resulting in 400 trap nights for each yearly capture event. All *Reithrodontomy*s spp (SMHM, *R*. *raviventris* and the sympatric western harvest mouse *R*. *megalotis*) were marked using a unique numerical fur clip pattern or ear tag to identify individual recaptures before release at the site of capture [43]. All other captured rodents were identified to species, sex, and fur clipped to identify recaptures. Trapping occurred at the same location every year from 2000 to 2017 during June, except for 2001 (July) 2002 and 2003 (August).

### SECR modeling

We created capture histories for each unique SMHM captured during the 18-year study, then used the SECR package in R [46, 47] to obtain density estimates (D), detection functions (g0), and home range indices (σ) for each of the 18 years. Model selection began with comparing three detection functions (HN = half-normal, EX = negative exponential; HR = Hazard rate) to determine the best initial model. We then analyzed models where the baseline detection rate (g0; the probability of detecting an individual at its activity center) and the baseline home range index (σ) could vary depending on three covariates: 1) time, 2) behavior, and 3) individual effects, with individual effects represented in a finite mixture model with sex as the class. The time effect accounted for the detection function varying among capture occasions, while the behavioral effect considered the degree of boldness of SMHM towards traps after their first capture (i.e., “trap shy” or “trap happy”, where captured individuals may become subsequently less or more likely to enter a trap, respectively). Individual effects accounted for variation in detection and home range among male and female mice.

**Fig 2:**
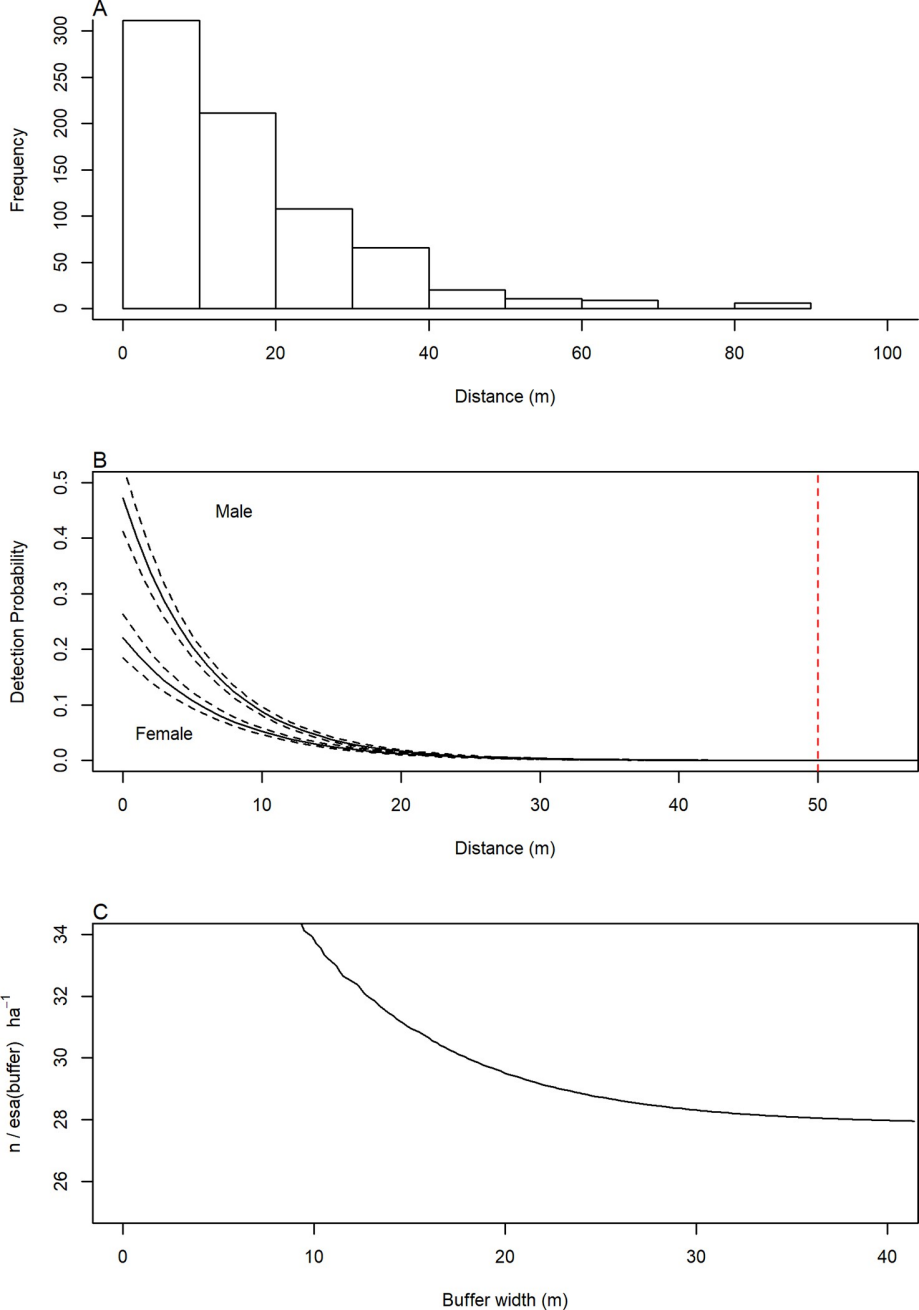
Three indicators inspected to determine the proper buffer distance to implement as well as to determine the correct detection function. A) Salt marsh harvest mouse frequency of distances moved in meters for the entire 18 year full 100 trap dataset; B) Detection probability curves for male and female SMHM related to distance (red line indicates buffer distance used); C) Effective sampling area as a function of increasing buffer width.

We estimated density within an explicit spatial sampling region. It included the trapping grid as well as a buffer sufficiently large to ensure all individuals were exposed to potential sampling. We used the values of σ obtained from a preliminary analysis to develop the spatial region in SECR, with the buffer around the trapping grid being based on a distance at least 4 times the initial σ parameter (50 m; Fig 2), which was also consistent with movement distances (11.9 m) found by Bias and Morrison 1999 [48]. We used the Akaike Information Criterion (AIC) [49] to determine the best fit model, which was the negative exponential detection function that allowed g0 and σ to vary by sex (Fig 2).

### Trapping variation analysis

To assess various reduced trapping effort, we used two methods; 1) We subsampled from our original trapping design and replicated the SECR modeling to produce new estimates of D, g0, and σ; and 2) We used the estimates of D, g0, and σ for the full 18 year dataset as the true values for Monte Carlo simulations. Monte Carlo simulation is a modeling approach commonly used to estimate the outcomes of subsampling scenarios. Here we evaluated reduced trap number, reduced number of sampling days and reduced trap density, using probability distributions derived from the full dataset [50].

**Fig 3:**
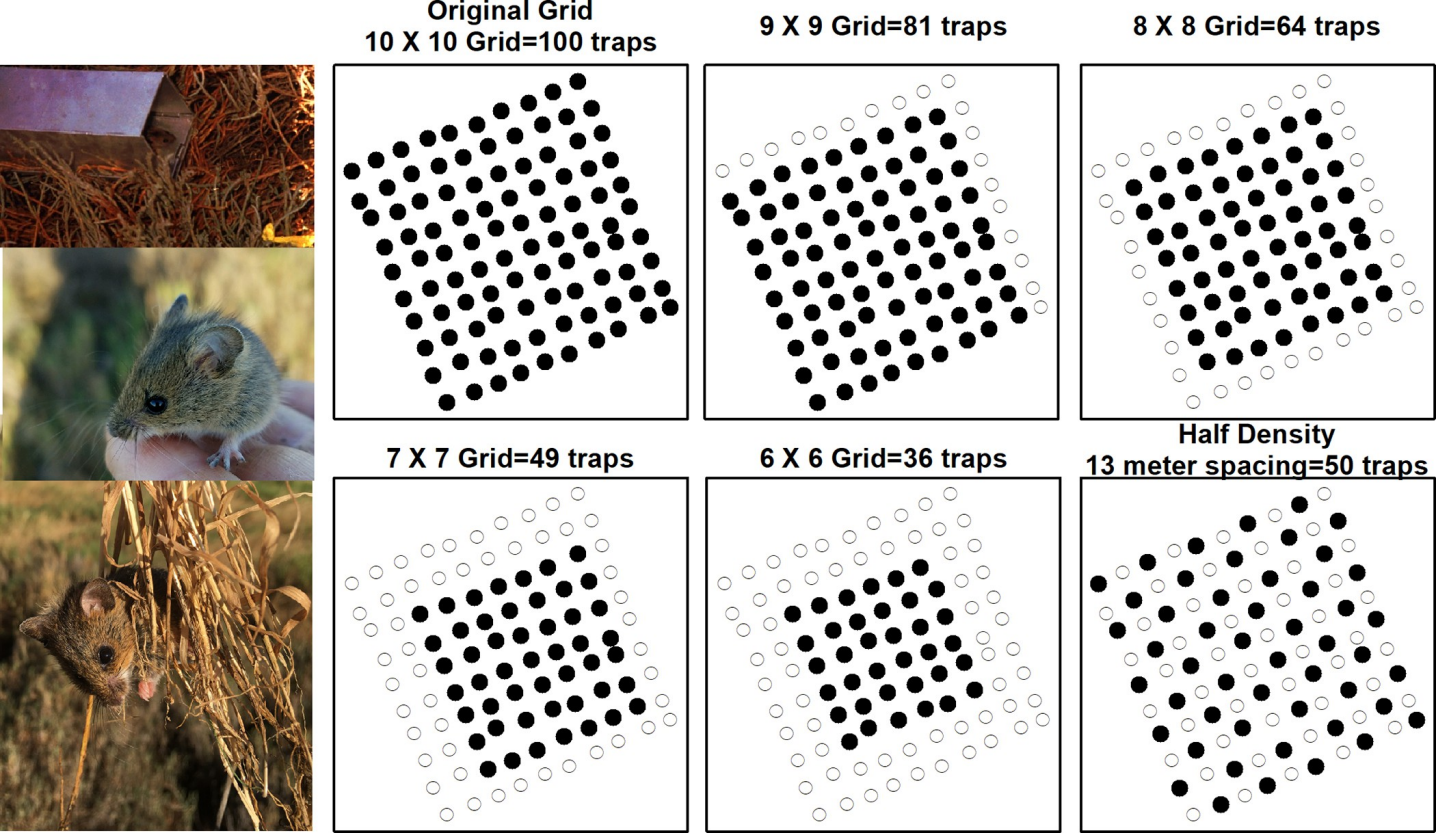
Crescent Unit trapping grid variations for subsampling reduced trapping designs. From top left to bottom right: Original field data collection layout of 10x10 grid, 9x9 grid, 8x8 grid, 7x7 grid, 6x6 grid, and a half density grid with 50 traps. Photos of small mammal trapping (photo credits: L. Thompson, I. Woo, and W. Thein).

Subsampling from our original 4-night 100 trap grid design included a 9x9 grid (81 traps), 8x8 grid (64 traps), 7x7 grid (49 traps), 6x6 grid (36 traps), 5x5 grid (25 traps), 4x4 grid (16 traps), a half density (50 traps) trapping design, and a 3-night trapping occasion (100 traps; Fig 3). The half density variation removed every other trap, creating a repeating five-dice pattern and resulted in a mean trap spacing of 13 meters calculated by the SECR package (Fig 3). SECR modeling was then completed for each of the subsamples for each year. This allowed us to compare density estimates for reduced trapping effort to the true density estimates on a yearly basis. Absolute difference and percent absolute difference were assessed for density, SE, g0, and σ to determine if a reduced sampling effort was comparable to the original design.

Monte Carlo simulations were performed with three variables being manipulated to assess their effects on density estimates: (1) trap number (16, 25, 36, 49, 64, 81, and 100); (2) number of consecutive trapping occasions (3 or 4); and (3) trap spacing (10 m, 15m, and 20m). These simulations provided 100 density estimates for comparison to the original trapping design. Performance of estimators was summarized as the average across replicates of relative bias (RB)

$${RB}_{j}=\frac{\left({\hat{D}}_{j}-D_{j}\right)}{D_{j}},$$ (1)

and root mean square error (RMSE)

$${RMSE}_{j}=\sqrt{\sum_{j=1}^{n}\frac{{\left({\hat{D}}_{j}-D_{j}\right)}^{2}}{n}}$$ (2)

where Dj is the true number in realization j

### Relative trapping effort

The relative trapping effort was expressed in terms of the staff time required to conduct each subsampling scenario was estimated relative to the full design as the baseline. Time estimates to conduct a complete survey included pre-survey setup and logistics (e.g., gathering and ensuring gear is in good working condition, mixing bait), survey time (e.g., setting and checking traps, processing animals), and post-survey trap cleaning.

## Results

### Trapping

Between 2000 and 2017, we captured 716 individual SMHM over 7,200 trap nights. Annual density estimates (individuals ha^-1^) ranged from 9.13±2.70 SMHM ha^-1^ in 2017 to 53.84±6.58 in 2005, with an overall mean density estimate of 30.76 SMHM ha^-1^ for the 18-year study period. Between 2000 and 2010, SMHM density estimates ranged between 16.88±3.65 and 53.84±6.58 with a mean density of 37.34 SMHM ha^-1^. Afterwards, between 2011 and 2017, SMHM density estimates seemed to stabilize at a lower density ranging between 9.13±2.70 and 28.39±4.72 with a mean of 20.41 SMHM ha^-1^.

### Trap number

**Fig 4:**
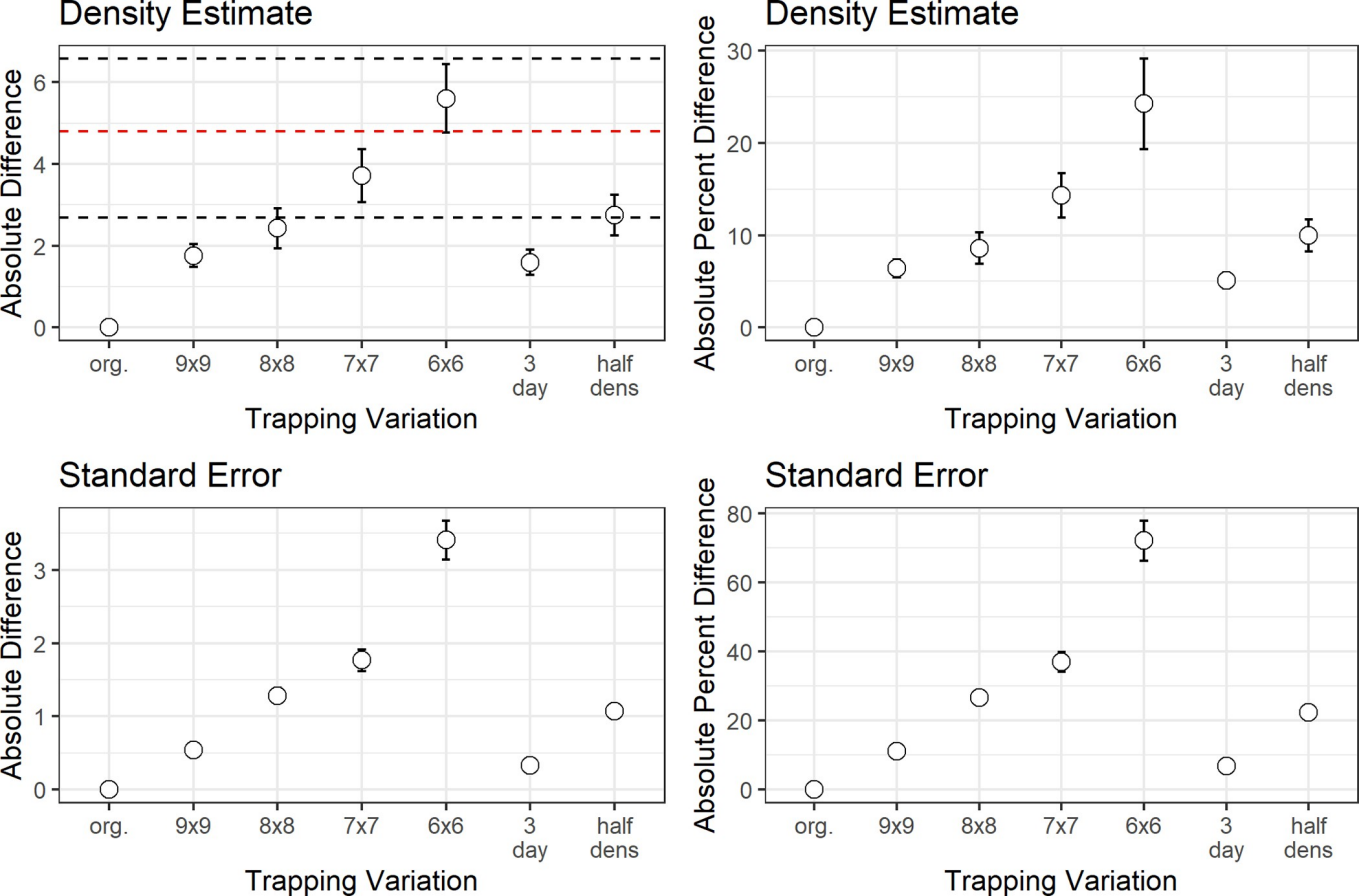
Absolute difference and absolute percent difference for both density estimates and standard error (SE) across years for the full dataset compared to the subsampled datasets. (lower black line indicates the minimum SE observed [2017], red line indicates the mean SE, and upper black line indicates the maximum SE observed [2005]).

Reducing the number of traps (while maintaining the original spacing interval) systematically reduced the number of total captures from 716 (full dataset) to 272 (6x6 grid; Table 1), which drastically reduced the number of data points on which the model was built. Further subsampling and reduction to the 5x5 (25 traps) and 4x4 (16 traps) grids resulted in extremely large deviations of model parameters and density estimates; therefore, these two designs will not be discussed further. Model parameters varied for the trap number subsampled datasets, with male g0 being lower for the reduced trap number variations except for the 6x6 grid (36 traps), and female g0 higher for all reduced trap number variations (S1 Table). Male σ remained relatively stable except for the 6x6 grid, in which it was smaller compared to the other trap number variations; female σ was also smaller for all subsampled trap number variations (S1 Table). These differences in model parameters led to differences in the model’s ability to replicate density estimates of the full dataset. For instance, a reduction from 100 traps to 81 traps led to a mean absolute difference in density estimates of 1.76 individuals per hectare (6.4% change), whereas a reduction to 36 traps led to a mean difference in density of 5.61 individuals per hectare (24.3% change; Table 1). The ability of the trap number models to replicate (within the SE of the full dataset) the full datasets density estimate varied across years with the 9x9 grid successfully replicating the full dataset 100 percent of the time, while the 6x6 grid (50%) was half as successful (Fig 4). Averaging across years, all trap number variations except the 6x6 grid stayed within the SE of the full dataset (Fig 4). To ensure there was no spatial bias from reducing the number of traps, we created five spatially different 7x7 grids and calculated density estimates for each across all years. All five grids resulted in density estimates not significantly different from each other across all years (F = 1.086, p = 0.369; S1 Fig)

**Table 1:** Salt marsh harvest mouse density estimates (mice/ha) and capture numbers [D(capture)] for the full dataset and subsampled datasets between 2000 and 2017.

		Area				Trap Spacing	Duration
Year	Full Dataset	9x9	8x8	7x7	6x6	Half-Density	3-day
2000	26.11 (34)	29.43 (31)	34.04 (28)	33.81 (23)	37.28 (18)	20.79 (19)	28.08 (33)
2001	32.03 (41)	31.39 (32)	31.19 (25)	24.19 (16)	27.33 (13)	33.55 (30)	32.39 (37)
2002	44.9 (58)	40.49 (42)	38.98 (32)	35.47 (24)	39.48 (19)	40.73 (37)	48.87 (56)
2003	26.93 (35)	24.88 (26)	27.13 (22)	26.84 (18)	33.51 (16)	21.82 (20)	25.57 (30)
2004	28.66 (37)	25.43 (26)	27.85 (22)	23.18 (15)	18.95 (9)	22.85 (21)	31.42 (36)
2005	53.84 (69)	55.59 (57)	58.57 (47)	53.35 (35)	63.26 (30)	60.62 (54)	50.04 (57)
2006	16.88 (22)	15.06 (16)	14.31 (12)	11.6 (8)	10.57 (5)	18.42 (17)	17.04 (20)
2007	53.26 (69)	55.45 (58)	51.55 (42)	53.36 (36)	54.46 (26)	50.95 (46)	52.61 (61)
2008	33.4 (43)	31.91 (33)	29.59 (24)	30.83 (21)	31.31 (15)	34.41 (31)	34.9 (40)
2009	51.85 (67)	51.88 (54)	52.04 (42)	49.38 (33)	50.69 (24)	51.8 (47)	55.08 (63)
2010	42.89 (55)	41.91 (43)	41.54 (33)	39.43 (26)	41.89 (20)	43.43 (39)	41.89 (48)
2011	15.6 (20)	15.42 (16)	15.04 (12)	16.57 (11)	20.94 (10)	18.04 (16)	16.68 (19)
2012	20.54 (27)	17.86 (19)	18.13 (15)	17.57 (12)	16.54 (8)	21.66 (20)	20.9 (25)
2013	21.63 (28)	23.14 (24)	24.42 (20)	19.55 (13)	25.13 (12)	27.28 (25)	22.48 (26)
2014	28.39 (37)	27.5 (29)	25.53 (21)	23.21 (16)	25.13 (12)	30.35 (28)	28.09 (33)
2015	23.73 (31)	26.45 (28)	26.4 (22)	29.18 (20)	37.28 (18)	24.73 (23)	21.87 (26)
2016	23.82 (31)	25.06 (26)	21.95 (18)	20.88 (14)	18.74 (9)	26.41 (24)	20.54 (24)
2017	9.13 (12)	8.58 (9)	9.62 (8)	11.6 (8)	16.54 (8)	9.72 (9)	9.29 (11)
Avg.	30.7 (39.7)	30.4 (31.6)	30.4 (24.7)	28.8 (19.3)	31.6 (15.1)	30.9 (28.1)	30.9 (35.8)
Total	553.6 (716)	547.4 (569)	547.8 (445)	520 (349)	569 (272)	557.6 (506)	557.7 (645)
Percent Change		-1.11% (-20.5%)	-1.03% (-37.8%)	-6.07% (-51.2%)	2.79% (-62.0%)	0.71% (-29.3%)	0.75% (-9.9%)

**Fig 5:**
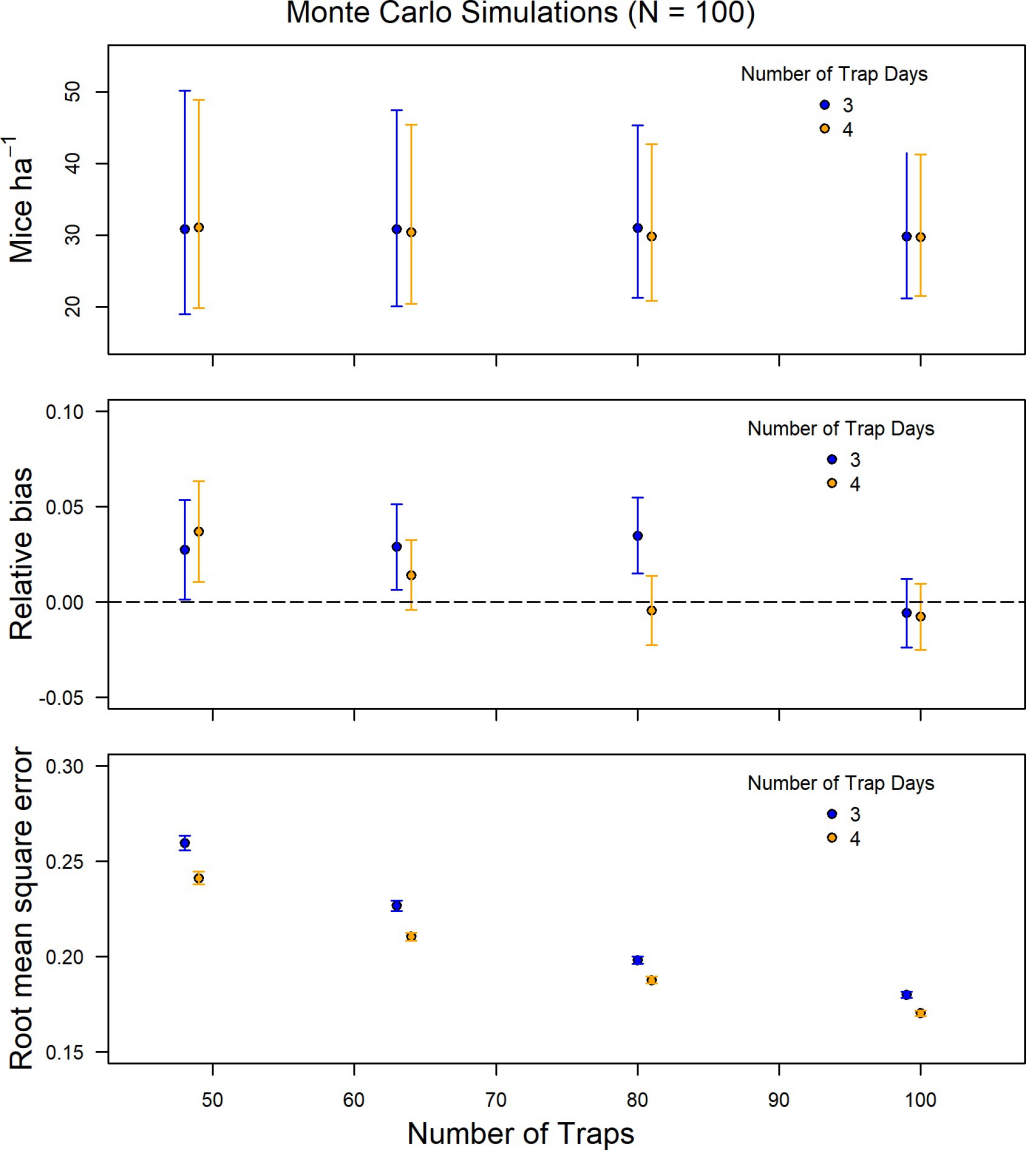
Density estimate, relative bias, and root mean square error for Monte Carlo simulations (n = 100) using the mean density, detection function, and home range as the true values for comparing the number of trapping occasions (3, 4), and the number of traps (100, 81, 64, 49).

The Monte Carlo simulations were consistent with the trap number subsampling results and indicated that a reduction in the number of traps did not affect density estimates when grids had more than 49 traps (7x7 grid; Fig 4). However, a reduction below 64 traps (8x8 grid) resulted in larger variance in RB and RMSE, thus reducing precision of the density estimates (Fig 5).

### Trap density

When reducing the density of the traps by 50 percent while maintaining the original sampling area (effectively increasing the trap spacing from 9.43 meters to 13 meters), the model maintained comparable trapping data with a 29 percent reduction in total captures (n = 506) when compared to the full dataset (n = 716; Table 1). Model parameters were also similar to the full dataset; male g0 had the largest change with a reduction in detection, and female g0 was slightly higher (S1 Table). However, both male and female σ remained relatively stable though slightly smaller than the full dataset, and sex ratio did not change (S1 Table). These small differences in model parameters led to the model’s ability to replicate similar density estimates of the full dataset. The reduction from 100 traps to 50 traps with 13 meter spacing led to a mean absolute difference in density estimates of 2.75 individuals per hectare (9.9% change; Table 2). The ability of the trap spacing model to replicate (within the SE of the full dataset) the full datasets density estimate varied across years, but in general successful replication of the full dataset occurred 72 percent of the time (Fig 4).

**Table 2:** Difference of density estimates for the subsampled datasets compared to the full dataset, showing mean, absolute mean, and absolute max summaries.

Year	Full Dataset	Difference from the Full Data set					
		9x9	8x8	7x7	6x6	Half-Dens.	3-day
2000	26.11	-3.32	-7.94	-7.71	-11.17	-1.98	5.31
2001	32.03	0.63	0.84	7.84	4.70	-0.36	-1.52
2002	44.91	4.41	5.93	9.44	5.42	-3.97	4.17
2003	26.93	2.04	-0.20	0.08	-6.58	1.36	5.11
2004	28.66	3.23	0.80	5.47	9.70	-2.76	5.81
2005	53.84	-1.75	-4.73	0.49	-9.42	3.79	-6.78
2006	16.89	1.82	2.57	5.28	6.31	-0.16	-1.53
2007	53.26	-2.20	1.71	-0.11	-1.20	0.65	2.31
2008	33.40	1.49	3.80	2.57	2.09	-1.50	-1.01
2009	51.85	-0.04	-0.19	2.46	1.15	-3.23	0.04
2010	42.90	0.98	1.35	3.46	1.00	1.00	-0.54
2011	15.61	0.18	0.57	-0.96	-5.34	-1.08	-2.44
2012	20.55	2.68	2.41	2.98	4.00	-0.36	-1.11
2013	21.64	-1.51	-2.79	2.08	-3.50	-0.85	-5.65
2014	28.40	0.89	2.86	5.18	3.26	0.31	-1.96
2015	23.74	-2.71	-2.67	-5.44	-13.55	1.86	-1.00
2016	23.83	-1.24	1.87	2.94	5.08	3.29	-2.59
2017	9.13	0.55	-0.49	-2.47	-7.41	-0.16	-0.59
mean		0.34	0.32	1.87	-0.86	-0.23	-0.22
Absolute mean		**1.76**	2.43	3.72	5.61	**2.75**	**1.59**
Absolute max		4.41	7.94	9.44	13.55	6.78	**3.97**

**Fig 6:**
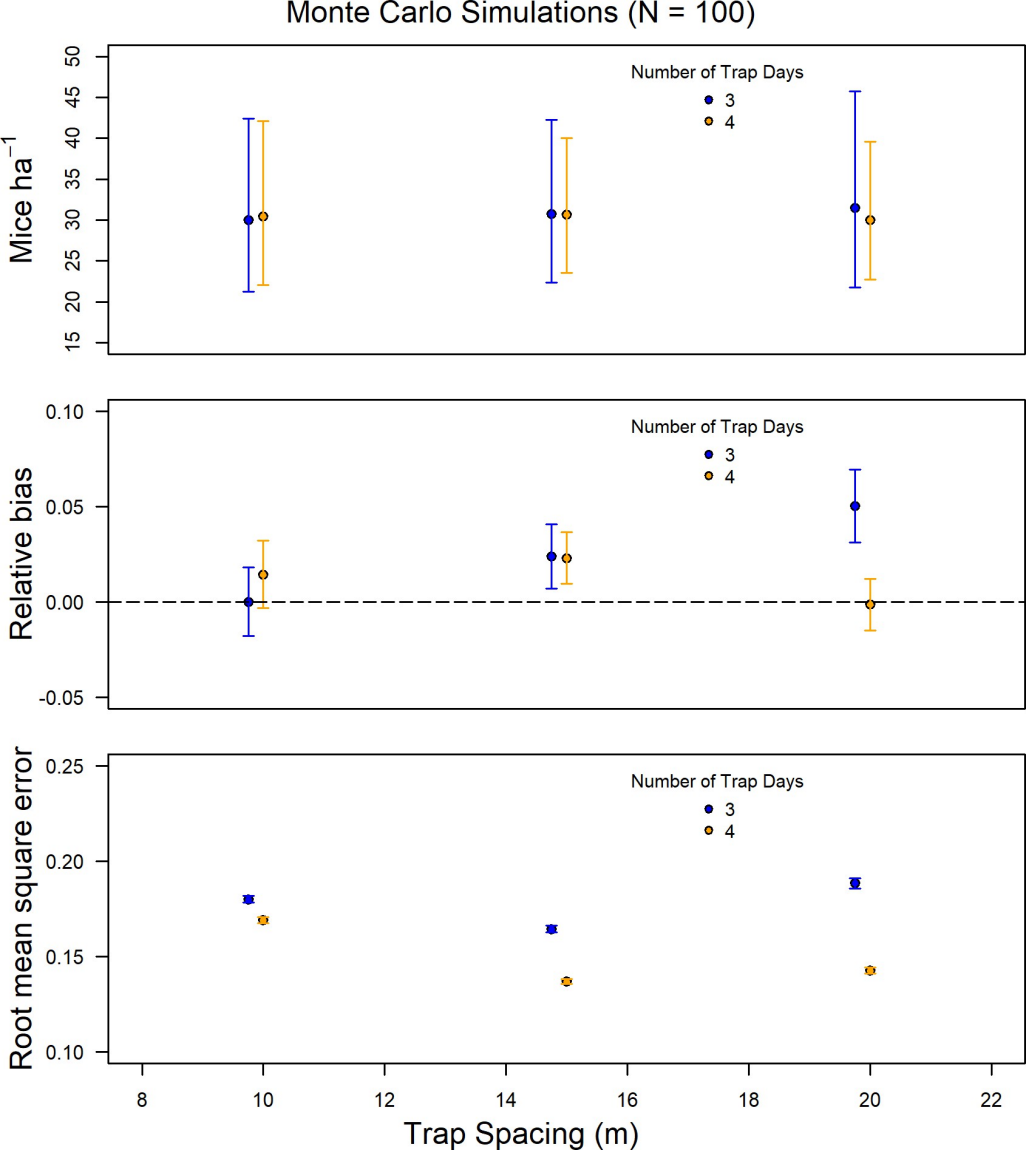
Density estimate, relative bias, and root mean square error for Monte Carlo simulations (n = 100) using the mean density, detection funtion, and home range as the true values for comparing the number of trapping occasions (3, 4), and trap spacing (10, 15, 20 meters).

The Monte Carlo simulations were consistent with trap spacing subsampled data, showing that an increase in trap spacing from 10 m to 15 m or 20 m did not affect density estimates or RB and actually reduced RMSE (Fig 6).

### Trapping duration

With reduced trapping duration from 4 nights to 3 nights, the model maintained comparable trapping data with only a 9.9 percent reduction in total captures (n = 645) when compared to the full dataset (n = 716; Table 1). Model parameters varied from the full dataset with male g0 being slightly less and σ being slightly larger than the full dataset (S1 Table). However, female parameters had large changes with g0 increasing and σ decreasing (S1 Table). Despite these rather large changes in model parameters, the model maintained the ability to replicate density estimates within 1 SE of the full dataset. The reduction from 4 nights to 3 nights led to a mean absolute difference in density estimates of 1.59 individuals per hectare (5.09% change; Table 2). Reducing the trap nights from 4 nights to 3 nights resulted in model agreement with the full dataset across all years and, averaging across years, the density estimates stayed within the SE of the full dataset (Fig 4).

Monte Carlo simulations also showed that a reduction to a 3-night trapping occasion design resulted in similar estimates of density and RB as in the full dataset with only a small increase in the RMSE (Figs 5 and 6). The only instances when reduction of trapping duration adversely affected the model’s ability to replicate density, RB, and RMSE, was when combined with a reduction in trapping numbers or with an increase in trap spacing (Figs 5 and 6).

### Overall trapping variation

Subsampling scenarios reduced the number of captures and therefore the ability of the model to accurately replicate the detection rate, home range, and sex ratio of the full dataset (S1 Table). This reduction in the accuracy of model parameters propagated in the model’s ability to replicate density estimates. Due to the reduction in the number of traps, fewer individuals were captured (as well as recaptured; Table 1), and density estimates in the subsampled datasets were less precise than those in the full dataset (Fig 4). Overall, the 9x9 trap grid as well as the 3-night trapping subsampling scenarios remained within one standard error of the density estimate for the full datasets in all 18 years, whereas the other subsample designs were less likely to achieve this accuracy: 8x8 (94%), half-density (77%), 7x7 (61%), and 6x6 (50%; Fig 4). Across all years, the 9x9 trapping grid and 3-night trapping occasion designs had less than a 2 individuals per hectare difference from the original design, and the half-density and 8x8 trapping grid designs were both under 3 individuals per hectare (Fig 5).

Overall, correlations among raw capture numbers, capture efficiency, and, especially, differences in the original density estimates and density estimates of the reduced study designs indicated a trapping duration of 3-nights and a 9x9 grid arrangement were the least biased modifications of the original design. Correlations (*r*) among the original density estimates and density estimates of the reduced study designs were generally high (range in *r* = 0.89 to 0.99; S2 Fig). But the ranges in annual bias estimates for the 3-night trapping duration and 9x9 grid arrangement were at least half those for the other grid and trap spacing arrangements (S3 Fig).

### Relative trapping effort

The reduction from four trapnights down to three trapnights resulted in the greatest time saved compared to the original full design, while all other subsampling scenarios resulted in minor time savings (<15%; Table 3). The relative percent time saved is estimated as a range, since processing fewer traps and animals would result in time savings; however preparations, logistics, and post survey cleaning efforts would be minimally impacted.

**Table 3:** Relative percent time saved with each subsampling scenario compared to the full grid design. Percent time estimates included pre-survey setup and logistics, time to conduct the surveys, and post-survey trap cleaning and data entry.

	Original	Subsampling Scenarios					
Grid Design	10x10 Grid	9x9 Grid	8x8 Grid	7x7 Grid	6x6 Grid	Half-Density	10x10 Grid
Number of Trap nights	4	4	4	4	4	4	3
Percentage of Time Saved	0%	0–5%	0–5%	5–10%	5–10%	10–15%	15–20%

## Discussion

### Population monitoring

Monitoring wildlife populations and their habitat is one of the most fundamental needs in wildlife management; however, managers face persistent challenges associated with accuracy, costs, and logistics, all of which play a role in implementing a long-term monitoring program and its sustainability into the future. Long-term monitoring of small mammal populations provides insights on population trends, can help determine if conservation measures are effective, and can inform managers whether further management actions are necessary for the recovery of an endangered species or to prevent biodiversity loss [51]. In particular, long-term datasets can be analyzed to relate population responses to different environmental factors, changes in landcover, or climatic changes (i.e., prolonged drought, severe storm surges) [e.g., 52–54] that may become more frequent in the future [55]. However, tracking population trends on small mammals can be arduous given their secretive nature and the difficulty in locating or counting individuals.

We leveraged one of the longest continuous monitoring datasets for the endangered SMHM and modeled the tradeoffs among subsampling approaches with reduced sampling area, sampling density, and number of sampling days. In terms of a reduced sampling area, the 9x9 grid was the only grid reduction pattern that resulted in density estimates within one SE of the original method for all 18 years and may be a viable option for reducing staffing effort while not biasing density estimates. Reducing the length of trapping from four to three nights resulted in density estimates within one SE of the original methods for the full 18-year dataset, but reducing sampling density to half the number of original traps resulted in density estimates that were within 1 SE of the original methods for only 14 of the 18 years (77%), indicating this sampling design could result in undesirable errors. Conard et al. (2008) examined the effect of trap density and trapping duration on species richness estimates and found that higher trap densities generally reached stable richness estimates in fewer nights than low density trapping arrangements [56]. They found that at least 3 nights duration was needed to produce a stable estimate of species richness for the range of trap densities tested (9–144 trap stations/ha), indicating the importance of carefully selecting duration and density parameters to meet research objectives [56].

### SECR modeling

Wildlife monitoring using live trapping is an important management tool to inform conservation objectives and management actions, especially for species that are difficult to detect [57, 58]. Estimating abundance from a SECR model can lead to greater flexibility and efficiency in study design [59]. Our results suggest application of SECR models provides a viable approach to identify trapping designs that save time and maintain the accuracy needed to compare trends from long-term monitoring, especially for protected species. Sollmann, et al. (2012) [18] showed that, while there are limits to the flexibility in spatial trap design for SECR modelling, it proved more robust to changes in trapping area size and spacing relative to animal movement than non-spatial capture-recapture models. We found differences in parameter model estimates for subsampled datasets which may be due to individual differences in detection and movement that become more apparent when a smaller portion of the overall population is sampled.

Study designs should strive to sample as many individuals as possible to obtain adequate data on individual movement, leading to improved precision of parameter estimates. This precision may be particularly important for difficult to detect species, including threatened or endangered species that may have smaller populations or low trap encounter rates. A trapping design that is small relative to a species home range may result in the capture of too few individuals and reduce the ability of the model to estimate parameters [60]. For instance, reducing the area of the trap array to 0.50 ha (7x7 grid) had a large effect on sample size (sampling only 49% of the original individuals averaged across 18 years), and therefore, had a significant effect on estimates. Particularly, female SMHM detection rate was overestimated and home range was underestimated (S1 Table). Another important finding was that reducing the trapping area had a much larger effect than reducing the trap density. A reduction of traps by 50 percent (7x7 grid, 49 traps) reduced the individuals captured by 51 percent (n = 349), whereas the reduction in trap density from 10 meter spacing to 13 meter spacing (Half density, 50 traps) only reduced the individuals captured by 29 percent (n = 506). Therefore, the half density design maintained a larger proportion of the full dataset and resulted in density estimates with a mean percent difference of 9.98 compared to the 7x7 trap design which had density estimates with a mean percent difference of 14.35. This difference was most likely due to better model estimates for female g0 and σ (S1 Table, Fig 4) arising from the larger sample size. Efford and Fewster (2013) [61] showed that SECR estimates of realized population size were generally less biased than estimates from non-spatial methods and showed adequate precision. Furthermore, these estimates were shown to be robust for some severe deviations from the fitted model of home-range centers.

Many wildlife managers traditionally use CE as a quick way to determine population status from year to year [62, 63] (S2 Table). While CE does provide the ability to quickly look at a trend in the capture data, it only provides the number of unique individuals over the number of trap nights. Capture-recapture models consider how many times a unique individual is captured and when the individual was captured during the trapping event to create population estimates. SECR models also take into account where an individual was captured spatially to provide more robust density estimates [34, 57, 59]. This explicit spatial component of SECR models allows for better comparisons of real density estimates not only across years and space but also across trapping designs, ultimately leading to more informed sampling designs. Kristensen et al (2018), found that study design considerations will differ among species based on their habitat and movements, recommending collecting preliminary pilot trapping data to integrate into SECR simulations providing a means to optimize the study design for long term monitoring [64]. Our results also show that SECR methodology can be employed in the planning/monitoring of a wide array of species by maximizing effectiveness and minimizing cost and effort. SECR modeling is not only transferable across species but can be an important tool for managers implementing adaptive management approaches.

### Management applications

Common management challenges include balancing the need for effective and efficient long-term monitoring programs with available resources [62]. Financial and staffing constraints are important considerations that may determine the spatial and temporal scales of survey efforts in long-term monitoring projects [65–68], and reduced funding or other resources may create barriers to long-term data collection. Additionally, managers may have an interest in minimizing impacts to habitats, protected species, and co-occurring species. Constraints while trapping small mammals, in particular, include accessing protected/sensitive habitats and impacts to rare or endangered species [52, 57]; therefore, reducing field efforts can have benefits. Our study showed that some reduced trapping efforts can produce estimates comparable to those from more intensive efforts (e.g. our original 10x10 grid trapped for four consecutive nights), suggesting there may be opportunities to re-evaluate field efforts and gain efficiency. The reduction in staff time was greatest when reducing the trapping occasion from four down to three consecutive nights. Although the time savings overall were modest because of the pre- and post- survey logistics and requirements, a shorter consecutive trapping duration can provide considerable flexibility in scheduling small mammal trapping surveys, especially when environmental factors such as flooding from high tides, extreme weather, and hunting days can limit the days on which surveys can occur.

Despite the temporal extent of the dataset, we acknowledge that our analysis was based on a single grid. A SECR modeling approach with data from multiple grids and sites could incorporate small mammal variability from environmentally and ecologically distict areas and provide further insights for species monitoring and management. For example, if a species is present but captures are too low to derive meaningful density estimates, the minimum number of animals known alive (MNKA) can be used to derive an index to density. Deriving the relationship between density and MNKA would require data from multiple grids with an adequate number of captures as well as sites with few captures to assess density based differences in capture probabilities. From a management perspective, trapping effort commonly varies by site because of factors such as access, vegetation structure, and changing environmental constraints (e.g. high tide inundation). Evaluating different grid arrangements relative to these site factors would almost certainly provide managers with information that would allow them to better balance logistical constraints with collecting adequate data to meet the survey goals.

Long-term datasets, such as what we used here, provide an opportunity to evaluate sampling protocols and assess whether modifications might result in similar estimates and still meet the goals of the project. Reassessment of monitoring efforts may result from shifting monitoring or conservation goals [69, 70]. Concerns about funding, staff time, priorities, impacts to species or their habitats can result in reductions or losses of long-term monitoring efforts. Field sampling optimization exercises, such as what we present here, can provide a means to modify large scale monitoring practices to meet programmatic needs and operational constraints. This type of analysis can help managers understand how to optimize trapping efforts, such as potentially expanding monitoring to underrepresented but priority target habitat areas.

## Conclusion

Here, we use SECR modelling to illustrate that a reduction in the number of trapping nights, trap numbers, and trap density can provide small mammal density estimates similar to those from the original design. The application of SECR models can inform future species monitoring, reevaluation of current monitoring, and implementation of adaptive management strategies [71]. Wildlife managers concerned about the detection and trapping design for small mammals can leverage long-term datasets and SERC models to inform their methodologies. In addition, many endangered species are difficult to study due to small populations numbers, fragmented habitats, and their elusive nature; this can reduce confidence in population density trend estimates. SERC modeling can be a valuable tool in these situations. The analysis here showed how long-term monitoring datasets collected with a consistent and robust methodology can be used to understand how sampling designs might be modified to optimize management investments and still achieve similar results. The SERC approach is a powerful spatial modeling tool to estimate population densities and determine an optimal study design for long-term monitoring.

## Supporting information

S1 Table Detection rate, home range index and sex ratio parameters for the full dataset and subsampled datasets, and difference and percent difference for the subsampled datasets. (DOCX)

S2 Table Comparison of salt marsh harvest mouse density estimates and capture efficiency [D, (CE)] between the original and subsampled trapping variations. (DOCX)

S1 Fig Density estimates of five different spatial variations (Center[A], Northwest [B], Northeast [C], Southwest [D], Southeast [E]), showing no significant effect on density estimates.
(TIF)

S2 Fig Relationship between the density (individuals per ha) of salt marsh harvest mice (*Reithrodontomys raviventris*) derived from spatially explicit capture-recapture models for a 10 m x 10 m grid trapped over 4 consecutive days (full grid; x-axis) and density in arrangements with either: Smaller numbers of traps in the grid (9 x 9, 8 x 8, 7 x 7, 6 x 6); larger trap spacing (20 m, i.e. a grid with 50 traps); and shorter trapping duration (3 consecutive days). (TIFF)

S3 Fig Bias (%) in annual density estimates (individuals ha^-1^) of salt marsh harvest mice (*Reithrodontomys raviventris*) derived from spatially explicit capture-recapture models for a 10 m x 10 m grid trapped over 4 consecutive days (full grid; x-axis) with density in arrangements with either: Smaller numbers of traps in the grid (9 x 9, 8 x 8, 7 x 7, 6 x 6); larger trap spacing (20 m, i.e. a grid with 50 traps); and shorter trapping duration (3 consecutive days). Trapping was conducted once each year from 2000 through 2017.
(TIFF)
