# Supplementary material for: Assessing small-mammal trapping design using spatially explicit capture recapture (SECR) modeling on long-term monitoring data
Source: PLoS One. 2022 Jul 5;17(7):e0270082. doi: 10.1371/journal.pone.0270082 (PMC9255754; doi:10.1371/journal.pone.0270082)
Supplement: S1 Table — (DOCX) [file pone.0270082.s001.docx]

S1 Table. Detection rate, home range index and sex ratio parameters for the full dataset and subsampled datasets, and difference and percent difference for the subsampled datasets.

|  |  | **Detection Rate (g0)** | | **Sigma (home range Index)** | | **Sex ratio (M/F)** | **g0 and Sigma Combined Change** |
| --- | --- | --- | --- | --- | --- | --- | --- |
|  | **Variation** | **Male** | **Female** | **Male** | **Female** |  |  |
| **Parameters** | original | 0.47 | 0.22 | 5.96 | 6.91 | 0.53 | - |
|  | 9x9 | 0.42 | 0.24 | 6.17 | 6.25 | 0.51 | - |
|  | 8x8 | 0.4 | 0.24 | 6.03 | 6.04 | 0.51 | - |
|  | 7x7 | 0.45 | 0.26 | 6.08 | 6.08 | 0.5 | - |
|  | 6x6 | 0.49 | 0.25 | 5.07 | 6 | 0.51 | **-** |
|  | Half Density | 0.41 | 0.24 | 5.81 | 6.58 | 0.53 | **-** |
|  | 3-night | 0.45 | 0.28 | 6.26 | 5.99 | 0.54 | **-** |
| **Difference** | 9x9 | -0.05 | 0.02 | 0.21 | -0.66 | -0.02 | 0.94 |
|  | 8x8 | -0.07 | 0.02 | 0.07 | -0.87 | -0.02 | 1.03 |
|  | 7x7 | -0.02 | 0.04 | 0.12 | -0.83 | -0.03 | 1.01 |
|  | 6x6 | 0.02 | 0.03 | -0.89 | -0.91 | -0.02 | 1.85 |
|  | Half Density | -0.06 | 0.02 | -0.15 | -0.33 | 0 | 0.56 |
|  | 3-night | -0.02 | 0.06 | 0.3 | -0.92 | 0.01 | 1.30 |
| **Percent Difference** | 9x9 | -10.64 | 9.09 | 3.52 | -9.55 | -3.77 | 32.80 |
|  | 8x8 | -14.89 | 9.09 | 1.17 | -12.59 | -3.77 | 37.75 |
|  | 7x7 | -4.26 | 18.18 | 2.01 | -12.01 | -5.66 | 36.46 |
|  | 6x6 | 4.26 | 13.64 | -14.93 | -13.17 | -3.77 | 45.99 |
|  | Half Density | -12.77 | 9.09 | -2.52 | -4.78 | 0.00 | 29.15 |
|  | 3-night | -4.26 | 27.27 | 5.03 | -13.31 | 1.89 | 49.88 |
