# Supplementary material for: Assessing small-mammal trapping design using spatially explicit capture recapture (SECR) modeling on long-term monitoring data
Source: PLoS One. 2022 Jul 5;17(7):e0270082. doi: 10.1371/journal.pone.0270082 (PMC9255754; doi:10.1371/journal.pone.0270082)
Supplement: S2 Table — (DOCX) [file pone.0270082.s002.docx]

S2 Table. Comparison of salt marsh harvest mouse density estimates and capture efficiency [D, (CE)] between the original and subsampled trapping variations.

|  |  | **Area Reduction** | | | | **Trap Spacing** | **Duration** |
| --- | --- | --- | --- | --- | --- | --- | --- |
| **Year** | **Original** | **9x9** | **8x8** | **7x7** | **6x6** | **Half-Density** | **3-day** |
| 2000 | 26.11 (8.5) | 29.43 (9.56) | 34.04 (10.93) | 33.81 (11.73) | 37.28 (12.5) | 20.79 (9.5) | 28.08 (11) |
| 2001 | 32.03 (10.25) | 31.39 (9.87) | 31.19 (9.76) | 24.19 (8.16) | 27.33 (9.02) | 33.55 (15) | 32.39 (12.33) |
| 2002 | 44.9 (14.5) | 40.49 (12.96) | 38.98 (12.5) | 35.47 (12.24) | 39.48 (13.19) | 40.73 (18.5) | 48.87 (18.66) |
| 2003 | 26.93 (8.75) | 24.88 (8.02) | 27.13 (8.59) | 26.84 (9.18) | 33.51 (11.11) | 21.82 (10) | 25.57 (10) |
| 2004 | 28.66 (9.25) | 25.43 (8.02) | 27.85 (8.59) | 23.18 (7.65) | 18.95 (6.25) | 22.85 (10.5) | 31.42 (12) |
| 2005 | 53.84 (17.25) | 55.59 (17.59) | 58.57 (18.35) | 53.35 (17.85) | 63.26 (20.83) | 60.62 (27) | 50.04 (19) |
| 2006 | 16.88 (5.5) | 15.06 (4.93) | 14.31 (4.68) | 11.6 (4.08) | 10.57 (3.47) | 18.42 (8.5) | 17.04 (6.66) |
| 2007 | 53.26 (17.25) | 55.45 (17.9) | 51.55 (16.4) | 53.36 (18.36) | 54.46 (18.05) | 50.95 (23) | 52.61 (20.33) |
| 2008 | 33.4 (10.75) | 31.91 (10.18) | 29.59 (9.37) | 30.83 (10.71) | 31.31 (10.41) | 34.41 (15.5) | 34.9 (13.33) |
| 2009 | 51.85 (16.75) | 51.88 (16.66) | 52.04 (16.4) | 49.38 (16.83) | 50.69 (16.66) | 51.8 (23.5) | 55.08 (21) |
| 2010 | 42.89 (13.75) | 41.91 (13.27) | 41.54 (12.89) | 39.43 (13.26) | 41.89 (13.88) | 43.43 (19.5) | 41.89 (16) |
| 2011 | 15.6 (5) | 15.42 (4.93) | 15.04 (4.68) | 16.57 (5.61) | 20.94 (6.94) | 18.04 (8) | 16.68 (6.33) |
| 2012 | 20.54 (6.75) | 17.86 (5.86) | 18.13 (5.85) | 17.57 (6.12) | 16.54 (5.55) | 21.66 (10) | 20.9 (8.33) |
| 2013 | 21.63 (7) | 23.14 (7.4) | 24.42 (7.81) | 19.55 (6.63) | 25.13 (8.33) | 27.28 (12.5) | 22.48 (8.66) |
| 2014 | 28.39 (9.25) | 27.5 (8.95) | 25.53 (8.2) | 23.21 (8.16) | 25.13 (8.33) | 30.35 (14) | 28.09 (11) |
| 2015 | 23.73 (7.75) | 26.45 (8.64) | 26.4 (8.59) | 29.18 (10.2) | 37.28 (12.5) | 24.73 (11.5) | 21.87 (8.66) |
| 2016 | 23.82 (7.75) | 25.06 (8.02) | 21.95 (7.03) | 20.88 (7.14) | 18.74 (6.25) | 26.41 (12) | 20.54 (8) |
| 2017 | 9.13 (3) | 8.58 (2.77) | 9.62 (3.12) | 11.6 (4.08) | 16.54 (5.55) | 9.72 (4.5) | 9.29 (3.66) |
| Total | 553.6 (179) | 547.4 (175.53) | 547.8 (173.74) | 520 (177.99) | 569 (188.82) | 557.6 (253) | 557.7 (214.95) |
| Percent Change | | 1.11 (1.88) | 1.03 (2.88) | 6.07 (0.52) | 2.79 (5.52) | 0.71 (41.34) | 0.75 (20.11) |
