# Supplementary figures and images for: Assessing small-mammal trapping design using spatially explicit capture recapture (SECR) modeling on long-term monitoring data
Source: PLoS One. 2022 Jul 5;17(7):e0270082. doi: 10.1371/journal.pone.0270082 (PMC9255754; doi:10.1371/journal.pone.0270082)

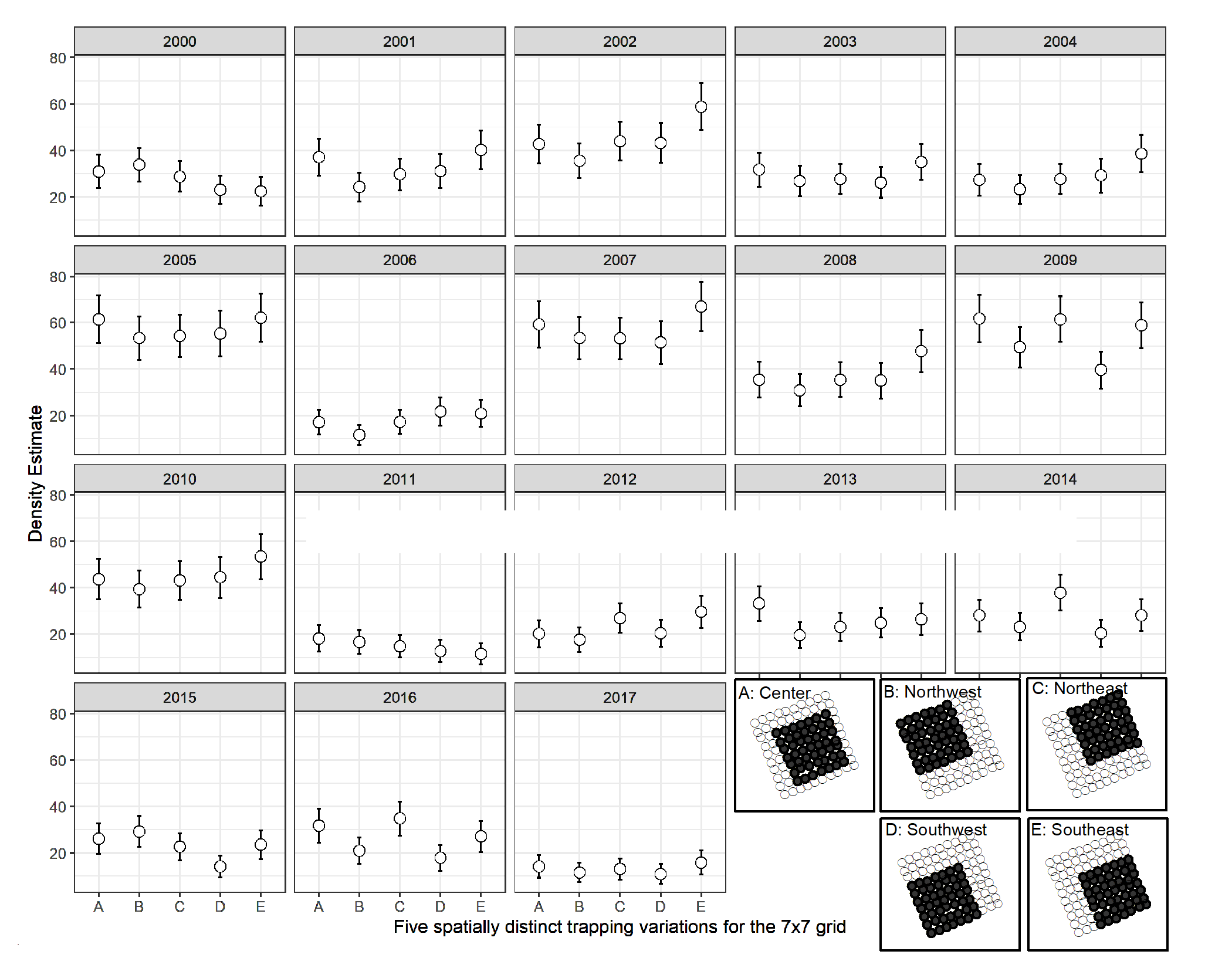

Supplement: S1 Fig — Density estimates of five different spatial variations (Center[A], Northwest [B], Northeast [C], Southwest [D], Southeast [E]), showing no significant effect on density estimates. (TIF) [file pone.0270082.s003.tif]

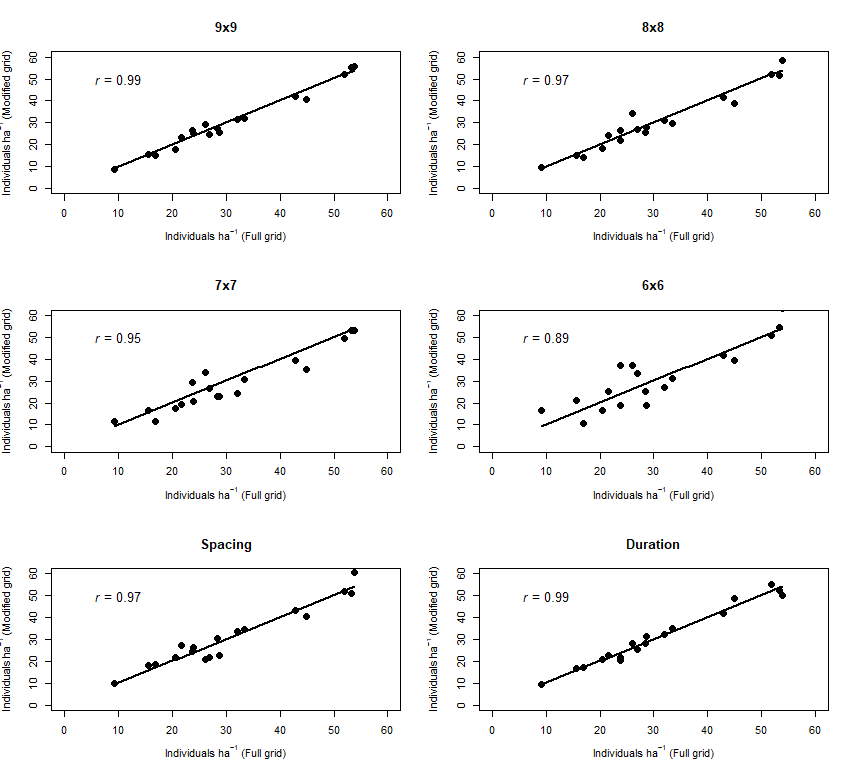

Supplement: S2 Fig — (TIFF) [file pone.0270082.s004.tiff]

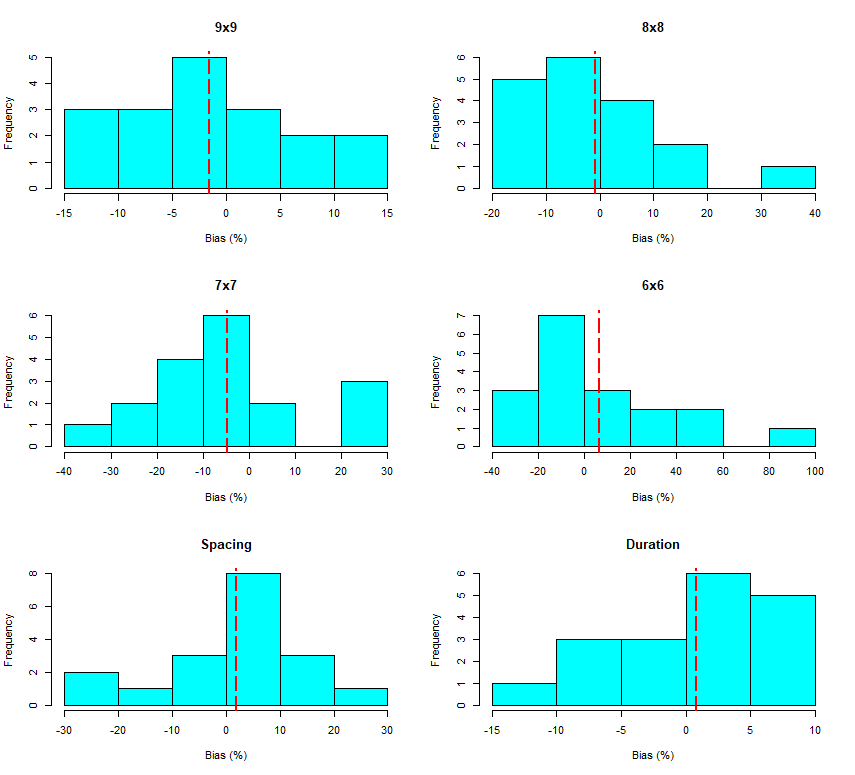

Supplement: S3 Fig — Trapping was conducted once each year from 2000 through 2017. (TIFF) [file pone.0270082.s005.tiff]
